# Multimodal deep learning for predicting WHO/ISUP grading in renal tumors on CT using a self-attention-based model: variable Vision Transformer (vViT)

**DOI:** 10.1016/j.ejro.2026.100796

**Published:** 2026-07-04

**Authors:** Takuma Usuzaki, Eriya Matsuno, Takashi Shizukuishi, Ryusei Inamori, Yuwen Zeng, Xiaoyong Zhang, Sota Oguro, Noriyasu Homma, Kei Takase

**Affiliations:** a Department of Diagnostic Radiology, Tohoku University Hospital, Sendai, Japan; b Department of Diagnostic Radiology, Japanese Red Cross Ishinomaki Hospital, Miyagi, Japan; c Department of Radiological Imaging and Informatics, Graduate School of Medicine, Japan

**Keywords:** World Health Organization/International Society of Urological Pathology (WHO/ISUP), Renal cell carcinoma (RCC), Deep learning, Machine learning, Vision transformer

## Abstract

**Purpose:**

To investigate the performance of the variable vision transformer (vViT) model in predicting the World Health Organization/International Society of Urological Pathology (WHO/ISUP) grade in renal tumors using multimodal data, including clinical information, radiomic features, and computed tomography (CT) images, and to identify the most dominant factor among these inputs.

**Materials and Methods:**

The vViT model was trained and validated on 111 patients (1398 images) and 15 patients (224 images), respectively, to classify renal tumors into low (WHO/ISUP grades 1–2) and high (grades 3–4) grades. The trained model was tested on 30 patients (228 images). The model inputs included class token, patient demographics (age, sex, body mass index), comorbidities (peripheral vascular disease, diabetes mellitus, chronic kidney disease), habits (smoking, alcohol use), radiomics, and CT images. Permutation feature importance was used to evaluate the contribution of each input sector. The model's performance was compared with other models; ViT, ConvNeXt, and ResNeXt using the DeLong test.

**Results:**

vViT achieved an accuracy of 0.811 and an area under the receiver operating characteristic curve (AUC-ROC) of 0.856 for the test dataset and outperformed ViT and ResNeXt (p < 0.05). In both predictions, the radiomic features were identified as the most dominant predictor of WHO/ISUP grade (p < 0.05).

**Conclusion:**

The performance of vViT had no significant difference compared with other models in predicting the WHO/ISUP pathological grade in renal tumors using multimodal data. These results highlight the potential of integrating radiomic analysis with clinical data in grading of renal tumors.

## 1 Introduction

The World Health Organization/International Society of Urological Pathology (WHO/ISUP) grading system has been widely used for the prognostic assessment of renal cancers and has shown greater consistency and reproducibility than traditional grading systems [1], [2], [3]. Histopathologically, renal cell carcinoma (RCC) accounts for 90% of renal cancers, and 70% and 15%−20% of RCC cases are clear cell RCC (ccRCC) and papillary RCC (pRCC), respectively [4], [5]. The WHO/ISUP grading has mainly been verified for the prognosis of ccRCC and pRCC; however, the prognostic value has been indicated for several subtypes of RCC [6], [7]. The treatment and follow-up plan should be changed according to the WHO/ISUP grades [8], [9]. The American Urological Association guidelines suggest that patients with low-grade ccRCC may choose less aggressive surgical strategies [8], [9], which can help reduce surgical invasion and support the preservation and improvement of renal function post-surgery. However, the WHO/ISUP grading before surgery requires a percutaneous biopsy, which is an invasive procedure associated with risks of infection, bleeding, and tumor rupture [10], [11]. Thus, there is a need for noninvasive methods that can predict the WHO/ISUP grade of renal tumors before surgery [2], [10]. Several models have been developed to predict the pathological grade of ccRCC. Xu L. et al. [10]. utilized deep learning (DL) to forecast the Fuhrman grade of ccRCC, which was the predecessor of WHO/ISUP, using DL models along with an ensemble model. These findings highlight the potential of DL with computed tomography (CT) images for predicting the pathologic grade of ccRCC. Zhang H. et al. [2]. created a preoperative predictive nomogram that integrates tumor spectral CT parameters and clinical data to estimate the WHO/ISUP grade of ccRCC. These studies also revealed a common challenge, i.e., the difficulty of employing traditional DL approaches to handle complex and diverse multimodal inputs effectively. This limitation underscores the need for other methodologies capable of integrating and analyzing multimodal data sources to predict the WHO/ISUP grade of renal tumors.

To address these challenges, inspired by the Vision Transformer (ViT), the variable vision transformer (vViT) model was proposed [12], [13], [14], [15], [16], [17]. This model can process sequences of various dimensions. Transformer architectures, which were initially designed for language tasks, are particularly effective for integrating image and nonimage data because of their self-attention mechanisms that capture long-range dependencies and context better than traditional models [18]. This capability is crucial for analyzing multimodal data such as medical images. vViT can analyze inputs such as clinical data, medical images, and image-derived features independently. Combining vViT with feature importance analysis allows for the identification of predominant elements from a vast array of inputs, specifically pinpointing key factors among clinical data, medical images, and image-derived features that predict the pathological grade of renal tumors [15]. The present study has two main aims: first, to investigate the performance of vViT in predicting the WHO/ISUP grade of renal tumors by utilizing clinical information, medical images, and image-derived features; and second, to identify the most dominant factor in predicting the WHO/ISUP grade of renal tumors.

## 2 Materials and methods

### 2.1 Data collection

Data used in this cross-sectional study were obtained from the training set of the 2019 Kidney and Kidney Tumor Segmentation Challenge (C4KC-KiTS). In using this dataset, we followed The Cancer Image Archive data usage policy and restrictions [19]. We included all available 210 patients with demographics, clinical data, WHO/ISUP grading, and preoperative contrast-enhanced CT images from the datasets. The retrospective data collection was approved by the University of Minnesota Institutional Review Board under Study 1611M00821.

### 2.2 Dataset construction: exposure, outcome, exclusion criteria, and random selection

#### 2.2.1 Definitions of exposure and outcome

In the present study, we attempted to predict the WHO/ISUP grade using demographics (age, gender, and body mass index [BMI]), disease history (peripheral vascular disease, diabetes mellitus, and chronic kidney disease), habit (smoking history and alcohol use), tumor radiomics, and CT images. We defined WHO/ISUP grades 1 and 2 as low grades, and 3 and 4 as high grades as previously reported [2].

### 2.3 Radiomic feature extraction and image processing

**Fig. 1:**
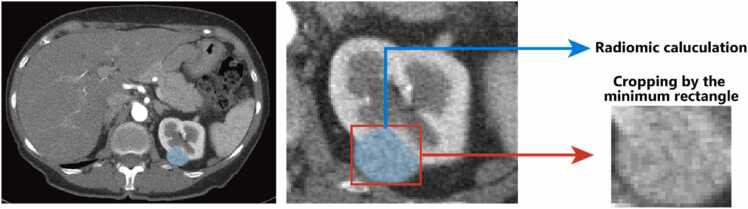
**The crop and calculation processes using segmentation.** The blue area shows the segmented renal tumor. Based on this segmentation, we determined the minimum rectangle to crop the renal tumor. The determined rectangle is shown in red. In addition, we calculated the radiomic features using the segmentation.

One hundred and five radiomic features (**Supplement 1**) were extracted using the PyRadiomics package [20]. The top 16 radiomic features ranked by F-value were selected from the training datasets in decreasing order. The selected features, F-scores, and p-values are shown in **Supplement 2**. Each tumor was cropped to the minimum rectangle that contained it. The cropped image was expanded to a 128 × 128 image by the Python Pillow package with the LANCZOS option. An example of the segmentation and cropping process is shown in Fig. 1.

### 2.4 Exclusion process and random selection for constructing datasets

**Fig. 2:**
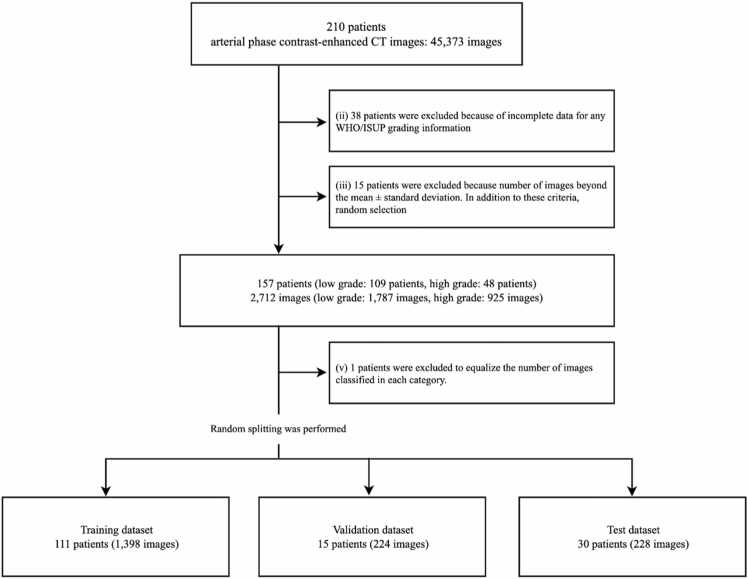
**The exclusion and selection procedures.** We excluded the patients with the exclusion criteria: (i) without data on age, gender, BMI, peripheral vascular disease, diabetes mellitus, chronic kidney disease, smoking history, or alcohol use information; (ii) without data on WHO/ISUP grading information; (iii) who had a number of images beyond the mean ± standard deviation; and (iv) who had radiomic features that could not be extracted from arterial phase contrast-enhanced CT images and whose annotation image of a tumor contained fewer than 256 pixels. Then, (v) random selection was performed to equalize the number of images per category to construct the training, validation, and test datasets.

We excluded patients (i) without data on age, gender, BMI, peripheral vascular disease, diabetes mellitus, chronic kidney disease, smoking history, or alcohol use information; (ii) without data on WHO/ISUP grading information; (iii) who had a number of images beyond the mean ± standard deviation; and (iv) who had radiomic features that could not be extracted from arterial phase contrast-enhanced CT images and whose annotation image of a tumor contained fewer than 256 pixels. We applied the selection criterion (iv) to exclude patients with extremely small tumors. After imposing these criteria, 157 patients (109 low grade, 48 high grade) with 2712 images (1787 low grade, 925 high grade) remained; no patient was excluded by (i), 38 patients were excluded by (ii), 15 patients were excluded by (iii), and no patients were excluded by (iv). In addition to these criteria, random selection (v) was used to equalize the number of images per category to construct the training, validation, and test datasets. After random selection, we obtained the training (111 patients [75 low grade, 36 high grade] with 1398 images [699 low grade, 699 high grade]), validation (15 patients [10 low grade, 5 high grade] with 224 images [112 low grade, 112 high grade]), and test (30 patients [23 low grade, 7 high grade] with 228 images [114 low grade, 114 high grade]) datasets. The selection and exclusion processes are summarized in Fig. 2.

### 2.5 Model architecture

**Fig. 3:**
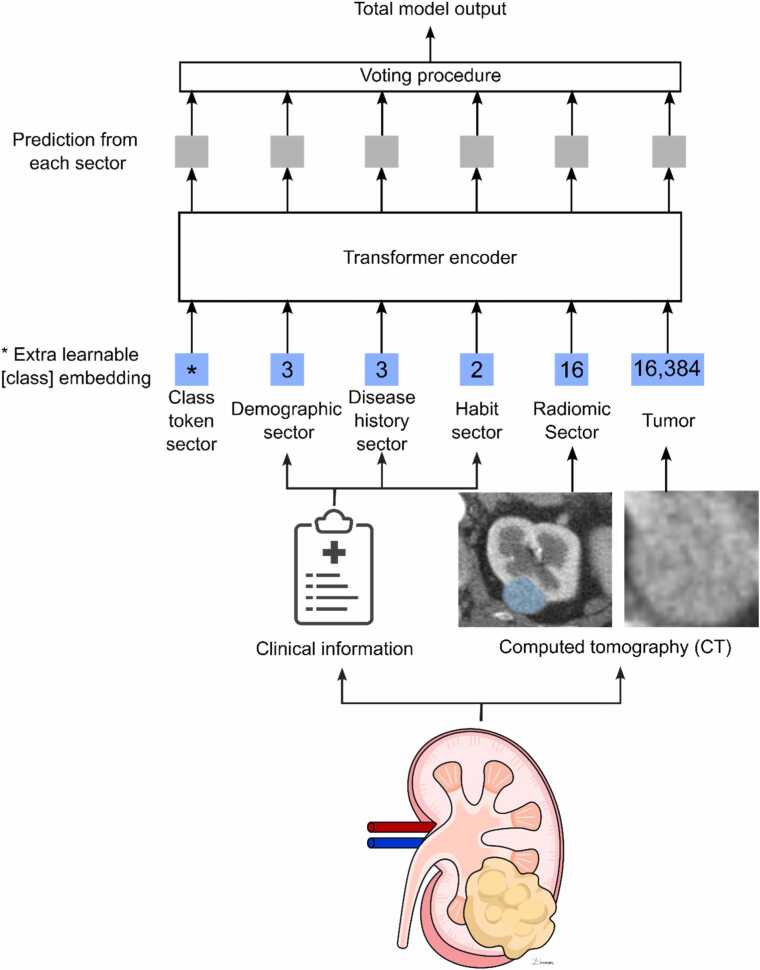
**Overview of the variable Vision Transformer (vViT) developed in this study.** The number in the blue rectangle represents the length of arrays input into vViT; demographics (2 features; age and sex, and body mass index [BMI]), disease history sector (peripheral vascular disease, diabetes mellitus, and chronic kidney disease), habit sector (smoking history and alcohol use), radiomic (16 selected features), and image sector. All arrays were converted to 1-dimentional arrays before inputting to vViT. The gray rectangles represent the prediction from each sector. The total model output was obtained by voting. Based on the original vViT we attached the class token sector.

We implemented vViT according to previous reports [12], [13], [14], [15], [16]. The detailed architecture of vViT and its mathematical explanation are shown in **Supplement 3**. Fig. 3 shows an overview of the vViT model architecture. In our implementation, the patch dimension, head dimension, number of heads, multi-layer perceptron dimension, and depth were 128, 64, 8, 32, and 8, respectively. We added a class token to the input as implemented in previous studies. The output from each sector was integrated into the total model output through the voting process. In vViT, the output from each sector can be derived as well as the total model output. Each compartment of the transformer and the classification head is termed a sector. The constructed vViT model had six sectors, including the class token sector: demographic sector (age, sex, and BMI), disease history sector (peripheral vascular disease, diabetes mellitus, and chronic kidney disease), habit sector (smoking history and alcohol use), radiomic sector, and image sector. Pytorch version 1.7.1 was used to implement vViT as the DL framework. Binary cross entropy was optimized by the Adam optimizer (*β*_1_=0.9, *β*_2_=0.999, *ε*=1.0 ×10^−8^, weight-decay=0, AMSGrad=False). In the training processes, we implemented augmentation to avoid overfitting using horizontal flip, vertical flip, perspective, invert, posterize, solarize, and equalize. The training process was repeated for 200 epochs.

### 2.6 Metrics and statistical analysis

#### 2.6.1 Metrics calculation

The characteristics of the patients and the calculated values are presented as the mean and 95% confidence interval (95%CI) or as the number (n) and ratio (%). We saved the models that achieved the best accuracy for the validation dataset. Next, metrics were calculated for the test dataset. We calculated the classification accuracy, sensitivity, specificity, positive predictive value (PPV), negative predictive value (NPV), F-score, logarithmic loss, and *κ* score using the model that achieved the best accuracy. Similarly, we calculated AUC-ROCs using the model that achieved the best AUC-ROC. As vViT was implemented to classify the patients by WHO/ISUP grade using images (image-based analysis), we organized the output of vViT to provide predictions for each patient (patient-based analysis). In this organizational approach, voting and mean were used for binary and continuous variables, respectively. The performance of image sector in vViT was compared with ViT [21], ConvNeXt [22], and ResNeXt [23]. These models were tested after 200 epochs of training using the same images as vViT. The model that achieved the highest accuracy for the validation dataset was saved. McNemar and DeLong tests were performed to compare the contingency Table and AUC-ROCs, respectively.

### 2.7 Permutation feature importance analysis

To evaluate the contribution of each sector to output, permutation feature importance was calculated by performing the following three calculation steps.

(i) Permutation was performed to the respective sector according to a previously reported method [24], [25], [26]. By this implementation, patient data were assigned to another patient.
(ii) After permutation was performed, accuracy was calculated using trained vViT.
(iii) Then, the difference between the original accuracy and that accuracy calculated using the permutated dataset was saved.

**Fig. 4:**
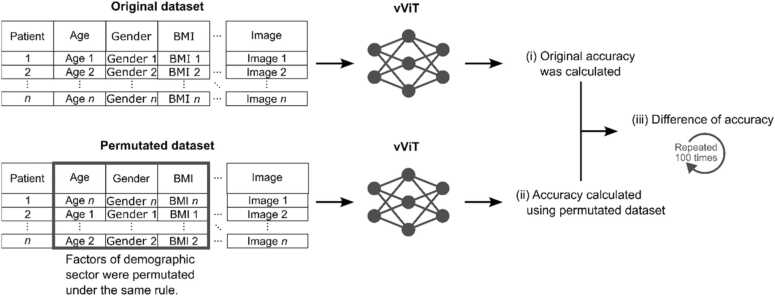
**Procedures of permutation importance analysis.** Demographics were permutated as an example. The following three steps were performed. (i) The original accuracy was calculated using the original dataset and trained variable vision transformer (vViT). (ii) Permutation was then performed, and accuracy was calculated using a permutated dataset and trained vViT. (iii) The difference between the original accuracy and that calculated using a permutated dataset was calculated. We defined the difference as the permutation importance. These processes were repeated 100 times by changing the permutation pattern.

Procedures (i), (ii), and (iii) were repeated 100 times for each sector. Fig. 4 shows the procedures for calculating the differences in accuracy, using the characteristics sector as an example. We compared the differences in accuracy for each sector using the Mann-Whitney *U* test. All analyses were performed using Python Language, version 3.8.2 (Python Software Foundation at http://www.python.org). Statistical significance was evaluated by 95%CI or p < 0.05.

## 3 Results

Table 1 shows the characteristics of patients for the training, validation, and test datasets.

**Table 1:** Patient characteristics.

Parameter	Training dataset	Validation dataset	Test dataset
	111 patients	15 patients	30 patients
	1398 images	224 images	228 images
Mean age (95%CI), years	59.8 (33.9–85.7)	56.9 (29.6–84.1)	59.8 (36.4–83.2)
Gender, n (%)			
Male	73 (65.8)	12 (80.0)	19 (63.3)
Female	38 (34.2)	3 (20.0)	11 (36.7)
BMI (95%CI), kg/m^2^	30.7 (18.7–42.7)	32.3 (19.2–45.4)	31.4 (17.7–45.1)
WHO/ISUP grade note^a^, n (%)			
High grade	75 (67.6)	10 (66.7)	23(76.7)
Low grade	36 (32.4)	5 (33.3)	7 (23.3)
Pathological diagnosis, n (%)			
Clear cell RCC	94 (84.7)	15 (100)	24 (80.0)
Papillary RCC	15 (13.5)	0 (0.0)	5 (16.7)
Others^b^	2 (1.8)	0 (0.0)	1 (3.3)
Comorbidity, n (%)			
Peripheral vascular disease	5 (4.5)	2 (13.3)	1 (3.3)
Diabetes mellitus	25 (22.5)	2 (13.3)	6 (20.0)
Chronic kidney disease	7 (6.3)	1 (6.7)	5 (16.7)
Smoking, n (%)			
Never	54 (48.6)	4 (26.7)	9 (30.0)
Previous	37 (33.3)	6 (40.0)	18 (60.0)
Current	20 (18.1)	5 (33.3)	3 (10.0)
Alcohol use, n (%)			
Never or not in the last 3 months	49 (44.1)	7 (46.6)	9 (30.0)
Two or fewer daily	54 (48.6)	4 (26.7)	18 (60.0)
More than two daily	8 (7.3)	4 (26.7)	3 (10.0)
Images per patient, n (95%CI)	12.6 (10.7–14.5)	14.9 (9.86–20.0)	7.60 (5.11–10.1)

Abbreviations: 95%CI, 95% confidence interval; BMI, body mass index; WHO/ISUP, World Health Organization/International Society of Urological Pathology; RCC, renal cell carcinoma.

a We defined WHO/ISUP grades 1 and 2 as low grade, and 3 and 4 as high grade.

b The training dataset contained an RCC not otherwise specified (NOS) and a renal tumor that was diagnosed as urothelial carcinoma, and the test dataset contained a multilocular cystic renal neoplasm with low malignant potential. Although WHO/ISUP grading is not validated for some of these subtypes, we did not exclude these patients because WHO/ISUP grade was defined in the dataset.

### 3.1 Image-based analysis

**Fig. 5:**
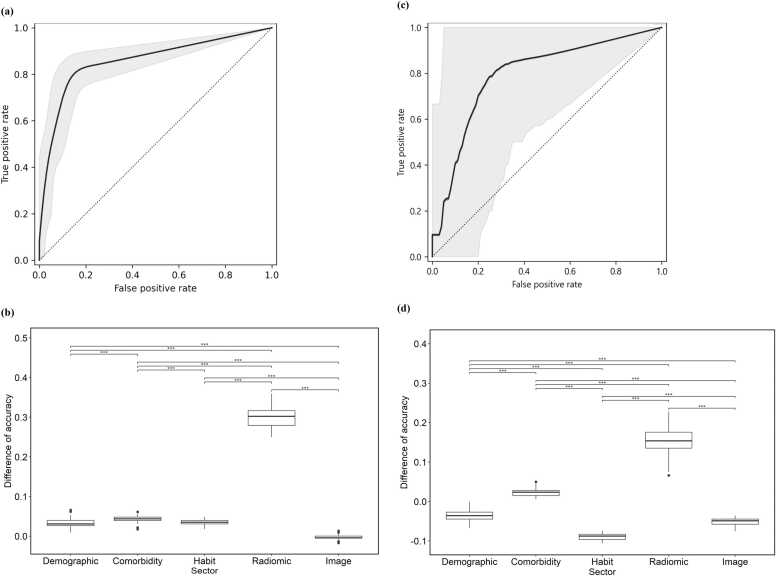
**Receiver operating characteristic (ROC) curves and results of the permutation importance analysis.** The ROC curves for the image- and patient-based analyses are shown in (a) and (c), respectively. The gray zone in each figure shows the 95% confidence interval (95%CI). The results of the permutation importance analysis for the image- and patient-based analyses are shown as boxplots in (b) and (d), respectively. Significance levels: * p < 0.05; ** p < 0.001; *** p < 0.0001.

Accuracy, sensitivity, specificity, PPV, NPV, F-score, AUC-ROC, logarithmic loss, and Cohen’s *κ* coefficients for the total model output for the test dataset were 0.811 (95%CI: 0.780–0.833), 0.833 (0.786–0.859), 0.789 (0.740–0.829), 0.798 (0.751–0.827), 0.826 (0.776–0.853), 0.815 (0.765–0.866), 0.856 (0.807–0.903), 3.63 (3.62–3.64), and 0.623 (0.597–0.648), respectively. Table 2**a** shows the metrics for each sector. Table 3**a** shows the performance of the CNN models and the results of the McNemar and DeLong tests. The image sector had no statistically different contingency tables compared with ViT (*p* = 0.11), ConvNeXt (0.65), and ResNeXt (0.74). The AUC-ROC of the image sector was higher than those of ViT and ResNeXt (*p* < 0.0001 for both). Sectors were ranked in descending order based on the permutation importance as radiomic (permutation importance=0.300, 95%CI: 0.248–0.352), comorbidity (0.0432, 0.0260–0.0605), habit (0.0341, 0.0215–0.0605), demographic (0.0332, 0.00956–0.0458), and image (−0.00290, −0.0187–0.0129) sectors. Figs. 5**a** and 5**b** show the ROCs and results of the permutation feature importance of each sector for the test dataset, respectively.

**Table 2:** Metrics of total model output and each sector.

**(a) Image-based analysis**							
Metrics (95%CI)	Total model	Class token sector	Demographic sector	Comorbidity sector	Habit sector	Radiomic sector	Image sector
Accuracy	0.811 (0.780–0.833)	0.794 (0.762–0.816)	0.838 (0.807–0.857)	0.820 (0.789–0.841)	0.794 (0.762–0.816)	0.798 (0.766–0.820)	0.816 (0.784–0.837)
Sensitivity	0.833 (0.786–0.859)	0.798 (0.749–0.828)	0.789 (0.740–0.820)	0.833 (0.786–0.859)	0.877 (0.832–0.898)	0.868 (0.822–0.891)	0.737 (0.687–0.771)
Specificity	0.789 (0.740–0.820)	0.789 (0.740–0.820)	0.886 (0.841–0.906)	0.807 (0.758–0.836)	0.711 (0.660–0.747)	0.728 (0.678–0.763)	0.895 (0.850–0.914)
PPV	0.798 (0.751–0.827)	0.791 (0.742–0.821)	0.874 (0.825–0.896)	0.812 (0.764–0.840)	0.752 (0.706–0.783)	0.762 (0.716–0.792)	0.875 (0.823–0.898)
NPV	0.826 (0.776–0.853)	0.796 (0.747–0.826)	0.808 (0.762–0.835)	0.829 (0.780–0.856)	0.853 (0.800–0.878)	0.847 (0.795–0.873)	0.773 (0.728–0.803)
F-score	0.815 (0.765–0.866)	0.795 (0.742–0.847)	0.829 (0.781–0.878)	0.823 (0.773–0.872)	0.810 (0.759–0.861)	0.811 (0.761–0.862)	0.800 (0.748–0.852)
κ score	0.623 (0.597–0.648)	0.588 (0.561–0.614)	0.675 (0.652–0.699)	0.640 (0.615–0.665)	0.588 (0.561–0.614)	0.596 (0.570–0.623)	0.632 (0.606–0.657)
AUC-ROC	0.856 (0.807–0.903)	0.825 (0.763–0.880)	0.855 (0.803–0.906)	0.859 (0.809–0.909)	0.858 (0.808–0.908)	0.862 (0.812–0.910)	0.861 (0.808–0.910)
Logarithmic loss	3.63 (3.62–3.64)	4.00 (4.00–4.01)	3.72 (3.71–3.72)	3.15 (3.14–3.16)	3.21 (3.21–3.22)	3.12 (3.11–3.12)	1.40 (1.39–1.41)
**(b) Patient-based analysis**							
Metrics (95%CI)	Total model	Class token sector	Demographic sector	Comorbidity sector	Habit sector	Radiomic sector	Image sector
Accuracy	0.733 (0.617–0.797)	0.767 (0.649–0.824)	0.767 (0.649–0.824)	0.800 (0.681–0.851)	0.667 (0.554–0.742)	0.667 (0.554–0.742)	0.767 (0.649–0.824)
Sensitivity	0.857 (0.535–0.927)	0.857 (0.535–0.927)	0.857 (0.535–0.927)	0.857 (0.535–0.927)	0.857 (0.535–0.927)	0.857 (0.535–0.927)	0.714 (0.431–0.846)
Specificity	0.696 (0.560–0.775)	0.739 (0.600–0.810)	0.739 (0.600–0.810)	0.783 (0.640–0.844)	0.609 (0.482–0.705)	0.609 (0.482–0.705)	0.783 (0.640–0.844)
PPV	0.462 (0.316–0.625)	0.500 (0.338–0.662)	0.500 (0.338–0.662)	0.545 (0.364–0.703)	0.400 (0.279–0.562)	0.400 (0.279–0.562)	0.500 (0.322–0.678)
NPV	0.941 (0.757–0.963)	0.944 (0.768–0.964)	0.944 (0.768–0.964)	0.947 (0.778–0.966)	0.933 (0.731–0.959)	0.933 (0.731–0.959)	0.900 (0.738–0.933)
F-score	0.600 (0.425–0.775)	0.632 (0.459–0.804)	0.632 (0.459–0.804)	0.667 (0.498–0.835)	0.545 (0.367–0.724)	0.545 (0.367–0.724)	0.588 (0.412–0.764)
κ score	0.426 (0.352–0.499)	0.478 (0.410–0.545)	0.478 (0.410–0.545)	0.534 (0.472–0.595)	0.333 (0.249–0.418)	0.333 (0.249–0.418)	0.432 (0.370–0.495)
AUC-ROC	0.795 (0.594–0.962)	0.770 (0.549–0.955)	0.752 (0.517–0.951)	0.764 (0.552–0.952)	0.714 (0.505–0.909)	0.714 (0.509–0.903)	0.801 (0.590–0.968)
Logarithmic loss	1.90 (1.84–1.96)	2.75 (2.70–2.81)	2.75 (2.69–2.81)	2.75 (2.69–2.81)	3.15 (3.09–3.21)	3.16 (3.10–3.21)	0.724 (0.666–0.782)

Abbreviations: 95%CI, 95% confidence interval; PPV, positive predictive value; NPV, negative predictive value; AUC-ROC, area under the receiver operating characteristic curve.

**Table 3:** Comparison of vViT with CNN.

**(a) Image-based comparison**				
Metrics (95%CI)	vViT (Image sector)	ViT	ConvNeXt	ResNeXt
Accuracy	0.816 (0.784–0.837)	0.644 (0.611–0.674)	0.776 (0.744–0.800)	0.767 (0.734–0.790)
Sensitivity	0.737 (0.687–0.771)	0.509 (0.461–0.556)	0.711 (0.660–0.747)	0.675 (0.625–0.714)
Specificity	0.895 (0.850–0.914)	0.781 (0.731–0.812)	0.842 (0.795–0.867)	0.858 (0.811–0.882)
PPV	0.875 (0.823–0.898)	0.699 (0.638–0.742)	0.818 (0.765–0.847)	0.828 (0.773–0.857)
NPV	0.773 (0.728–0.803)	0.614 (0.570–0.652)	0.744 (0.698–0.776)	0.724 (0.678–0.757)
F-score	0.815 (0.765–0.866)	0.589 (0.525–0.653)	0.761 (0.705–0.816)	0.744 (0.687–0.801)
p-value	-	0.11	0.65	0.74
AUC-ROC	0.861 (0.808–0.910)	0.689 (0.619–0.759)	0.826 (0.770–0.883)	0.719 (0.653–0.785)
p-value	-	< 0.0001	0.069	< 0.0001
**(b) Patient-based comparison**				
Metrics (95%CI)	vViT (Image sector)	ViT	ConvNeXt	ResNeXt
Accuracy	0.767 (0.649–0.824)	0.600 (0.492–0.685)	0.733 (0.617–0.797)	0.733 (0.617–0.797)
Sensitivity	0.714 (0.431–0.846)	0.286 (0.154–0.569)	0.714 (0.431–0.846)	0.714 (0.431–0.846)
Specificity	0.783 (0.640–0.844)	0.696 (0.560–0.775)	0.739 (0.600–0.810)	0.739 (0.600–0.810)
PPV	0.500 (0.322–0.678)	0.222 (0.128–0.483)	0.455 (0.297–0.636)	0.455 (0.297–0.636)
NPV	0.900 (0.738–0.933)	0.762 (0.612–0.831)	0.895 (0.727–0.930)	0.895 (0.727–0.930)
F-score	0.588 (0.412–0.764)	0.250 (0.0951–0.405)	0.556 (0.378–0.733)	0.556 (0.378–0.733)
p-value	-	1.0	1.0	1.0
AUC-ROC	0.801 (0.590–0.968)	0.721 (0.508–0.933)	0.808 (0.608–1.00)	0.708 (0.446–0.971)
p-value	-	0.43	0.78	0.22

Abbreviations: 95%CI, 95% confidence interval; vViT, variable Vision Transformer; ViT, Variable Transformer; PPV, positive predictive value; NPV, negative predictive value; AUC-ROC, area under the curve of the receiver operating characteristic.

### 3.2 Patient-based analysis

Accuracy, sensitivity, specificity, PPV, NPV, F-score, AUC-ROC, logarithmic loss, and Cohen's *κ* coefficients for the total model output for the test dataset were 0.733 (95%CI: 0.617–0.797), 0.857 (0.535–0.927), 0.696 (0.560–0.775), 0.462 (0.316–0.625), 0.941 (0.757–0.963), 0.600 (0.425–0.775), 0.795 (0.594–0.962), 1.90 (1.84–1.96), and 0.426 (0.352–0.499), respectively. Table 2**b** shows the statistics for each sector. Table 3**b** shows the performance of the CNN models and the results of the McNemar and DeLong tests. The image sector had no statistically different contingency tables compared with ViT (p = 1.0), ConvNeXt (1.0), and ResNeXt (1.0). Similarly, no statistically significant difference in the AUC-ROC of the image sector was found between the image sector of vViT and the other models. Sectors were ranked in descending order based on the permutation importance as radiomic (permutation importance=0.154, 95%CI: 0.0947–0.213), comorbidity (0.0226, 0.00467– 0.0406), demographic (−0.0359, −0.0613 to −0.0106), image (−0.0514, −0.0678 to −0.0351), and habit (−0.0892, −0.103 to −0.0757) sectors. Figs. 5**c** and 5**d** show the ROCs and the results of the permutation feature importance of each sector for the test dataset, respectively.

## 4 Discussion

The present study aimed to investigate the performance of vViT in predicting the WHO/ISUP pathological grade in renal tumors using multimodal data, including clinical information, radiomic features, and CT images. Additionally, we sought to identify the dominant factor among these input variables by combining vViT with permutation importance analysis. To the best of our knowledge, this is the first study to apply vViT for predicting the WHO/ISUP grade in renal tumors, and to determine the relative importance of clinical, radiomic, and imaging data in this context. In the image-based analysis, vViT achieved an accuracy of 0.811 and an AUC-ROC of 0.856 for the test dataset. vViT achieved a relatively lower accuracy of 0.733 and AUC-ROC of 0.795 in the patient-based compared with the image-based analysis. This lowering may be caused by a smaller sample size, selection bias, and multiple 2D-slice analyses. Moreover, high logarithmic loss in image-based (3.63) and patient-based (1.90) analyses indicates the limited sample size in training, validation, and test processes. Zhang H. et al. [2]. created a nomogram through multivariate logistic regression analysis, integrating spectral CT parameters and clinical data to predict high-grade ccRCC. This resulted in an AUC of 0.933, sensitivity of 0.810, and specificity of 0.923, with age, SII, and CP-K identified as independent predictors. The inconsistency with the present findings may be the result of differences in the characteristics of the study population and classification method. Hence, the reproducibility of each method should be continuously confirmed. Although analyzing only ccRCC or pRCC may simplify the problem setup, this setting might not accurately reflect real clinical conditions. We included RCC NOS, a renal tumor that was diagnosed as urothelial carcinoma, and a multilocular cystic renal neoplasm with low malignant potential. The utility of the WHO/ISUP grading has been indicated to RCC NOS and multilocular cystic renal neoplasms with low malignant potential [6], [7].

Notably, the image sector of vViT outperformed ViT and ResNeXt in AUC-ROC on the image-based analysis. This suggests that the self-attention mechanism and multimodal integration capabilities of vViT may offer advantages over image-only analyses by transformer and CNN architectures in predicting WHO/ISUP grades. However, the differences in performance between vViT and other models, was not statistically significant in patient-based analyses. Nonetheless, at a minimum, the present findings indicate that vViT is not inferior to transformer and traditional CNN architectures. The ability of vViT to handle sequences of varying dimensions and capture long-range dependencies through self-attention mechanisms likely contributed to its performance. Further research with larger datasets is necessary to establish the superiority of vViT and the potential in clinical use.

By leveraging permutation feature importance analysis [15], we were able to rank the input factors and identify the radiomic features as the most dominant predictors of ccRCC grade, followed by clinical factors such as comorbidities and demographics. The negative results of the permutation importance analysis indicate that raw CT images may degrade prediction performance. This degradation may be caused by 2D analysis. In the 2D analysis, we had to include slices that contained only a small part of the tumor, which may confuse parameter adjustment. To avoid this, selecting a key image in 2D, 2D projection from 3D images, and 3D analyses can be performed [27]. Quantitative analysis of tumor characteristics using radiomics provides greater predictive value than raw image data alone. By extracting high-dimensional features that quantify tumor shape, intensity, and texture, radiomics can uncover subtle patterns that may not be readily appreciable to the human eye [18]. Our results support the utility of integrating radiomic analysis with clinical data for improved WHO/ISUP grading.

The present study has some limitations. First, the retrospective single-center design may limit the generalizability of our findings. Future multi-institutional studies with prospective validation are needed to confirm the performance and transferability of vViT across different patient populations and imaging protocols. Moreover, to overcome the lack of clinical relevance in our study, comparison with a human expert should be considered. Second, the sample size, particularly for the test dataset in the patient-based analysis, was relatively small, which could have influenced the reliability of the results. Larger cohorts would provide more robust estimates of model performance and importance rankings. Moreover, we imposed the criteria (iii) to exclude large and small tumors, which may cause selection bias. We examined the performance of vViT under purified conditions. Third, this study exclusively involved patients who had undergone surgery, without considering the utility of vViT for patients who have had only biopsies. This omission may limit the applicability of our findings to the broader patient population who may undergo biopsy as a part of their diagnostic or treatment pathway. Fourth, the WHO/ISUP grading used in this study does not conform to the latest criteria, which could affect the relevance and applicability of our results in light of current diagnostic standards. Although several points were changed, there was limited changes in the main grading. We believe that vViT can be an effective model to predict WHO/ISUP grades. Finally, while permutation importance provides a useful overall ranking of input factors, it does not offer insights into the specific contributions of individual radiomic features. More granular analysis of the key radiomic variables driving the predictions would further elucidate the biological underpinnings and guide future radiomics research in WHO/ISUP grading.

## 5 Conclusion

We utilized vViT to predict the WHO/ISUP grade in renal tumors using multimodal data, including clinical information, radiomic features, and CT images. By combining vViT with permutation importance analysis, the input factors can be ranked based on their contribution to the prediction. The most dominant factor in predicting WHO/ISUP grade was radiomic features. The CT images themselves had relatively low permutation importance compared with the radiomic features derived from them. These findings suggest that the quantitative analysis of tumor characteristics through radiomics provides more predictive value than the raw image data alone.

## CRediT authorship contribution statement

**Eriya Matsuno:** Writing – review & editing, Writing – original draft. **Takuma Usuzaki:** Writing – review & editing, Writing – original draft, Visualization, Validation, Supervision, Software, Resources, Project administration, Methodology, Investigation, Funding acquisition, Formal analysis, Data curation, Conceptualization. **Ryusei Inamori:** Writing – review & editing, Writing – original draft. **Takashi Shizukuishi:** Writing – review & editing, Writing – original draft. **Kei Takase:** Writing – review & editing, Writing – original draft. **Noriyasu Homma:** Writing – review & editing, Writing – original draft. **Sota Oguro:** Writing – review & editing, Writing – original draft. **Xiaoyong Zhang:** Writing – review & editing, Writing – original draft. **Yuwen Zeng:** Writing – review & editing, Writing – original draft.

## Ethics approval and consent to participate

The University of Minnesota Institutional Review Board approved the retrospective data collection.

## Consent for publication

Not applicable.

## Funding

This work was supported by JSPS KAKENHI Grant Numbers 24K10803 and 24K10829.

## Declaration of Competing Interest

The authors have no relevant financial or non-financial interests to disclose.

## Data Availability

The datasets generated and/or analysed during the current study are available in the https://www.cancerimagingarchive.net/collection/c4kc-kits/#citations.

## References

[1] Delahunt B. (2009). Advances and controversies in grading and staging of renal cell carcinoma. Mod. Pathol..

[2] Zhang H., Li F., Jing M., Xi H., Zheng Y., Liu J. (2024). Nomogram combining pre-operative clinical characteristics and spectral CT parameters for predicting the WHO/ISUP pathological grading in clear cell renal cell carcinoma. Abdom. Radiol..

[3] Dagher J., Delahunt B., Rioux-Leclercq N., Egevad L., Srigley J.R., Coughlin G., Dunglinson N., Gianduzzo T., Kua B., Malone G., Martin B., Preston J., Pokorny M., Wood S., Yaxley J., Samaratunga H. (2017). Clear cell renal cell carcinoma: validation of world health organization/international society of urological pathology grading. Histopathology.

[4] Angori S., Lobo J., Moch H. (2022). Papillary renal cell carcinoma: current and controversial issues. Curr. Opin. Urol..

[5] Motzer R.J., Jonasch E., Agarwal N., Alva A., Baine M., Beckermann K., Carlo M.I., Choueiri T.K., Costello B.A., Derweesh I.H., Desai A., Ged Y., George S., Gore J.L., Haas N., Hancock S.L., Kapur P., Kyriakopoulos C., Lam E.T., Lara P.N., Lau C., Lewis B., Madoff D.C., Manley B., Michaelson M.D., Mortazavi A., Nandagopal L., Plimack E.R., Ponsky L., Ramalingam S., Shuch B., Smith Z.L., Sosman J., Dwyer M.A., Gurski L.A., Motter A. (2022). Kidney Cancer, Version 3.2022, NCCN clinical practice guidelines in oncology. J. Natl. Compr. Canc Netw..

[6] Delahunt B., Sika-Paotonu D., Bethwaite P.B., William Jordan T., Magi-Galluzzi C., Zhou M., Samaratunga H., Srigley J.R. (2011). Grading of clear cell renal cell carcinoma should be based on nucleolar prominence. Am. J. Surg. Pathol..

[7] Tsuzuki T. (2023). The latest pathological classification: WHO/ISUP. Jpn. J. Clin. Urol..

[8] Campbell S., Uzzo R.G., Allaf M.E., Bass E.B., Cadeddu J.A., Chang A., Clark P.E., Davis B.J., Derweesh I.H., Giambarresi L., Gervais D.A., Hu S.L., Lane B.R., Leibovich B.C., Pierorazio P.M. (2017). Renal mass and localized renal cancer: AUA guideline. J. Urol..

[9] S.C. Campbell, P.E. Clark, S.S. Chang, J.A. Karam, L. Souter, R.G. Uzzo, Renal Mass and Localized Renal Cancer: Evaluation, Management, and Follow-Up: AUA Guideline: Part I, J Urol 206(2) (2021) 199-208, ISSN = 1527-3792 (Electronic) 0022-5347 (Linking), DOI = 10.1097/JU.0000000000001911. 10.1097/JU.0000000000001911 34115547

[10] Xu L., Yang C., Zhang F., Cheng X., Wei Y., Fan S., Liu M., He X., Deng J., Xie T., Wang X., Liu M., Song B. (2022). Deep Learning Using CT images to grade clear cell renal cell carcinoma: development and validation of a prediction model. Cancers.

[11] Marconi L., Dabestani S., Lam T.B., Hofmann F., Stewart F., Norrie J., Bex A., Bensalah K., Canfield S.E., Hora M., Kuczyk M.A., Merseburger A.S., Mulders P.F.A., Powles T., Staehler M., Ljungberg B., Volpe A. (2016). Systematic review and meta-analysis of diagnostic accuracy of percutaneous renal tumour biopsy. Eur. Urol..

[12] Usuzaki T. (2022). Split. Expands Appl. range Vision. Transform. -- Var. Vision. Transform. (vViT).

[13] Usuzaki T., Inamori R., Ishikuro M., Obara T., Takaya E., Homma N., Takase K. (2024). Predicting EGFR status after radical nephrectomy or partial nephrectomy for renal cell carcinoma on CT using a self-attention-based model: variable vision transformer (vViT). J. Imaging Inform. Med..

[14] Usuzaki T., Inamori R., Shizukuishi T., Morishita Y., Takagi H., Ishikuro M., Obara T., Takase K. (2024). Predicting isocitrate dehydrogenase status among adult patients with diffuse glioma using patient characteristics, radiomic features, and magnetic resonance imaging: multi-modal analysis by variable vision transformer. Magn. Reson. Imaging.

[15] Usuzaki T., Takahashi K., Inamori R., Morishita Y., Shizukuishi T., Takagi H., Ishikuro M., Obara T., Takase K. (2024). Identifying key factors for predicting O6-Methylguanine-DNA methyltransferase status in adult patients with diffuse glioma: a multimodal analysis of demographics, radiomics, and MRI by variable Vision Transformer. Neuroradiology.

[16] Usuzaki T., Takahashi K., Inamori R., Morishita Y., Takagi H., Shizukuishi T., Toyama Y., Abe M., Ishikuro M., Obara T., Majima K., Takase K. (2024). Grading diffuse glioma based on 2021 WHO grade using self-attention-base deep learning architecture: variable Vision Transformer (vViT). Biomed. Signal. Process. Control..

[17] Usuzaki T., Takahashi K., Takagi H., Ishikuro M., Obara T., Inamori R., Kamada H., Sato T., Oguro S., Takase K. (2026). Performance of a self-attention-based model in the task of differentiating clear cell renal cell carcinoma from other renal tumors: variable Vision Transformer (vViT). Br. J. Radiol..

[18] Fuhrman S.A., Lasky L.C., Limas C. (1982). Prognostic significance of morphologic parameters in renal cell carcinoma. Am. J. Surg. Pathol..

[19] Clark K., Vendt B., Smith K., Freymann J., Kirby J., Koppel P., Moore S., Phillips S., Maffitt D., Pringle M., Tarbox L., Prior F. (2013). The Cancer Imaging Archive (TCIA): maintaining and operating a public information repository. J. Digit. Imaging.

[20] van Griethuysen J.J.M., Fedorov A., Parmar C., Hosny A., Aucoin N., Narayan V., Beets-Tan R.G.H., Fillion-Robin J.C., Pieper S., Aerts H. (2017). Computational radiomics system to decode the radiographic phenotype. Cancer Res..

[21] Dosovitskiy A., Beyer L., Kolesnikov A., Weissenborn D., Zhai X., Unterthiner T., Dehghani M., Minderer M., Heigold G., Gelly S., Uszkoreit J., Houlsby N. (2020). Image Is. Worth 16x16 Words Transform. Image Recognit. Scale.

[22] Z. Liu, H. Mao, C.-Y. Wu, C. Feichtenhofer, T. Darrell, S. Xie, A ConvNet for the 2020s, 2022, p. arXiv:2201.03545.

[23] S. Xie, R. Girshick, P. Dollár, Z. Tu, K. He, Aggregated Residual Transformations for Deep Neural Networks, 2016, p. arXiv:1611.05431.

[24] Usuzaki T., Ishikuro M., Murakami K., Noda A., Ueno F., Obara T., Kuriyama S. (2020). How can we evaluate whether an association is truly inter-generational?. J. Hypertens..

[25] Usuzaki T., Ishikuro M., Kikuya M., Murakami K., Noda A., Ueno F., Metoki H., Obara T., Kuriyama S. (2024). Child-parent associations of hematocrit in trios of Japanese adulthood confirmed by the random family method: The TMM BirThree Cohort Study. Sci. Rep..

[26] Usuzaki T., Chiba M., Shimoyama S., Hotta S. (2020). Random Fam. Method. Confirming Inter. -Gener. Relat. restricted re-Sampl..

[27] Yoon H., Kang D.Y., Kim S. (2024). Enhancement and evaluation for deep learning-based classification of volumetric neuroimaging with 3D-to-2D knowledge distillation. Sci. Rep..

