# Supplementary material for: Multimodal deep learning for predicting WHO/ISUP grading in renal tumors on CT using a self-attention-based model: variable Vision Transformer (vViT)
Source: Eur J Radiol Open. 2026 Jul 4;17:100796. doi: 10.1016/j.ejro.2026.100796 (PMC13355505; doi:10.1016/j.ejro.2026.100796)
Supplement: Supplementary file 1 — Supplementary material [file mmc1.pdf]

Supplement 1. The list of 105 radiomic features

First Order features (the number of features, 20)

The number of pixels, Energy, Total Energy, Entropy, Minimum, 10 Percentile, 90 Percentile, Maximum, Mean, Median, Range, Interquartile Range, Mean Absolute Deviation, Robust Mean Absolute Deviation, Root Mean Squared, Standard Deviation, Skewness, Kurtosis, Variance, and Uniformity.

Shape2D features (10)

Mesh Surface, Pixel Surface, Perimeter, Perimeter Surface Ratio, Sphericity, Spherical Disproportion, Maximum Diameter, Major Axis Length, Minor Axis Length, and Elongation.

Gray-Level Co-occurrence Matrix (GLCM) features (24)

Autocorrelation, Joint Average, Cluster Prominence, Cluster Shade, Cluster Tendency, Contrast, Correlation, Difference Average, Difference Entropy, Difference Variance, Joint Energy, Joint Entropy, Informational Measure of Correlation 1, Informational Measure of Correlation 2, Inverse Difference Moment, Maximal Correlation Coefficient, Inverse Difference Moment Normalized, Inverse Difference, Inverse Difference Normalized, Inverse Variance, Maximum Probability, Sum Average, Sum Entropy, and Sum Squares.

Gray Level Size Zone Matrix (GLSZM) features (16)

Small Area Emphasis, Large Area Emphasis, Gray Level Non Uniformity, Gray Level Non Uniformity Normalized, Size Zone Non Uniformity, Size Zone Non Uniformity Normalized, Zone Percentage, Gray Level Variance, Zone Variance, Zone Entropy, Low Gray Level Zone Emphasis, High Gray Level Zone Emphasis, Small Area Low Gray Level Emphasis, Small Area High Gray Level Emphasis, Large Area Low Gray Level Emphasis, and Large Area High Gray Level Emphasis.

Gray Level Run Length Matrix (GLRLM) features (16)

Short Run Emphasis, Long Run Emphasis, Gray Level Non Uniformity, Gray Level Non Uniformity Normalized, Run Length Non Uniformity, Run Length Non Uniformity Normalized, Run Percentage, Gray Level Variance, Run Entropy, Run Variance, Low Gray Level Run Emphasis, High Gray Level Run Emphasis, Short Run Low Gray Level Emphasis, Short Run High Gray Level Emphasis, Long Run Low Gray Level Emphasis, and Long Run High Gray Level Emphasis.

Neighboring Gray Tone Difference Matrix (NGTDM) features (5)

Busyness, Coarseness, Complexity, Contrast, and Strength.

Gray Level Dependence Matrix (GLDM) features (14)

Small Dependence Emphasis, Large Dependence Emphasis, Gray Level Non Uniformity, Dependence Non Uniformity, Dependence Non Uniformity Normalized, Gray Level Variance, Dependence Variance, Dependence Entropy, Low Gray Level Emphasis, High Gray Level Emphasis, Small Dependence Low Gray Level Emphasis, Small Dependence High Gray Level Emphasis, Large Dependence Low Gray Level Emphasis, and Large Dependence High Gray Level Emphasis.

Supplement 2. The list of selected radiomic features

Feature class	Feature	F-value	p-value
First Order	Entropy	304.49	<0.0001
GLCM	Contrast	368.19	<0.0001
	DifferenceAverage	381.39	<0.0001
	DifferenceEntropy	360.25	<0.0001
	DifferenceVariance	337.24	<0.0001
	Joint Entropy	361.65	<0.0001
	Idm	358.26	<0.0001
	Id	344.08	<0.0001
GLSZM	Zone Percentage	419.62	<0.0001
GLRLM	ShortRunEmphasis	324.35	<0.0001
GLRLM	RunLengthNonUniformityNormalized	365.18	<0.0001
GLRLM	RunPercentage	354.58	<0.0001
NGTDM	Contrast	369.46	<0.0001
GLDM	SmallDependenceEmphasis	396.96	<0.0001
GLDM	LargeDependenceEmphasis	312.74	<0.0001
GLDM	SmallDependenceHighGrayLevelEmphasis	362.13	<0.0001

Abbreviations: GLCM, Gray-Level Co-occurrence Matrix; GLSZM, Gray Level Size Zone Matrix; GLRLM, Gray Level Run Length Matrix; NGTDM, Neighboring Gray Tone Difference Matrix; GLDM, Gray Level Dependence Matrix.

Supplement 3. variable Vision Transformer (vViT) architecture

vViT receives a 1-dimensional sequence $\mathbf{x} \in \mathbb{R}$ and split sequences $\{a_n\}(n \in \mathbb{N}, a_n \in \mathbb{N}, 1 \leq n \leq N)$ as input. The input sequence $\mathbf{x}$ is divided into N sequences $\{\mathbf{x}_p^1, \mathbf{x}_p^2, \cdots \mathbf{x}_p^N\}$ according to split-sequence $\{a_n\}$: the length of $\mathbf{x}_p^n$ is equal to $a_n(1 \leq n \leq N)$. After splitting, the input sequence $\mathbf{x}$ is divided into N sequences $\{\mathbf{x}_p^1, \mathbf{x}_p^2, \cdots \mathbf{x}_p^N\}$, and linear transformation is performed by $\mathbf{D}_n \in R^{a_n \times m}$ to give the same dimension m to all patches. Similar to the `[class]` token in ViT, we prepend a learnable embedding to the sequence of embedded patches ($\mathbf{z}_0^0 = \mathbf{x}_{\text{class}}$), whose output values of the Transformer encoder ($\mathbf{z}_L^0$) represent the sequence $\mathbf{y}$. The classification head is implemented by a multilayer perceptron (MLP) with one hidden layer at the pretraining time and a single linear layer at the fine-tuning time based on a previous Transformer study. We prepend the embedded patches ($\mathbf{z}_0^0 = \mathbf{x}_{\text{class}}$) whose length is m. Transformer encoder comprises alternating layers of multiheaded self-attention and MLP blocks. Layernorm is applied before every block, and residual connections after every block. We implemented MLP using two layers with Gaussian Error Linear Unit nonlinearity. The classification head is implemented by an MLP with four hidden layers and a SoftMax function. By these implementations, vViT contains an arbitrary number of sectors that receive sequences of different lengths. The output from each sector was integrated by the voting process into the total model output. In vViT, the output from each sector can be derived as well as the total model output.

$$
\begin{aligned}
\mathbf{z}_{-1} &= [\mathbf{x}_p^1\mathbf{D}_1;\ \mathbf{x}_p^2\mathbf{D}_2;\ \cdots;\ \mathbf{x}_p^N\mathbf{D}_N] \quad \mathbf{D}_n \in \mathbb{R}^{a_n \times m} \\
\mathbf{z}_0 &= [\mathbf{x}_{\text{class}};\ (\mathbf{x}_p^1\mathbf{D}_1)\mathbf{E}_1;\ (\mathbf{x}_p^2\mathbf{D}_2)\mathbf{E}_2; \\
&\qquad\qquad \cdots;\ (\mathbf{x}_p^N\mathbf{D}_N)\mathbf{E}_N] + \mathbf{E}_{\text{pos}} \\
&\quad \mathbf{E}_n \in \mathbb{R}^{m \times d},\ \mathbf{E}_{\text{pos}} \in \mathbb{R}^{(N+1) \times d} \\
\mathbf{z}'_\ell &= \text{MSA}(\text{LN}(z_{\ell-1})) + z_{\ell-1} \quad \ell = 1, 2, \cdots, L, \\
\mathbf{z}_\ell &= \text{MLP}(\text{LN}(z'_\ell)) + z'_\ell \qquad \ell = 1, 2, \cdots, L, \\
\mathbf{y} &= \text{LN}(z_L^0).
\end{aligned}
$$

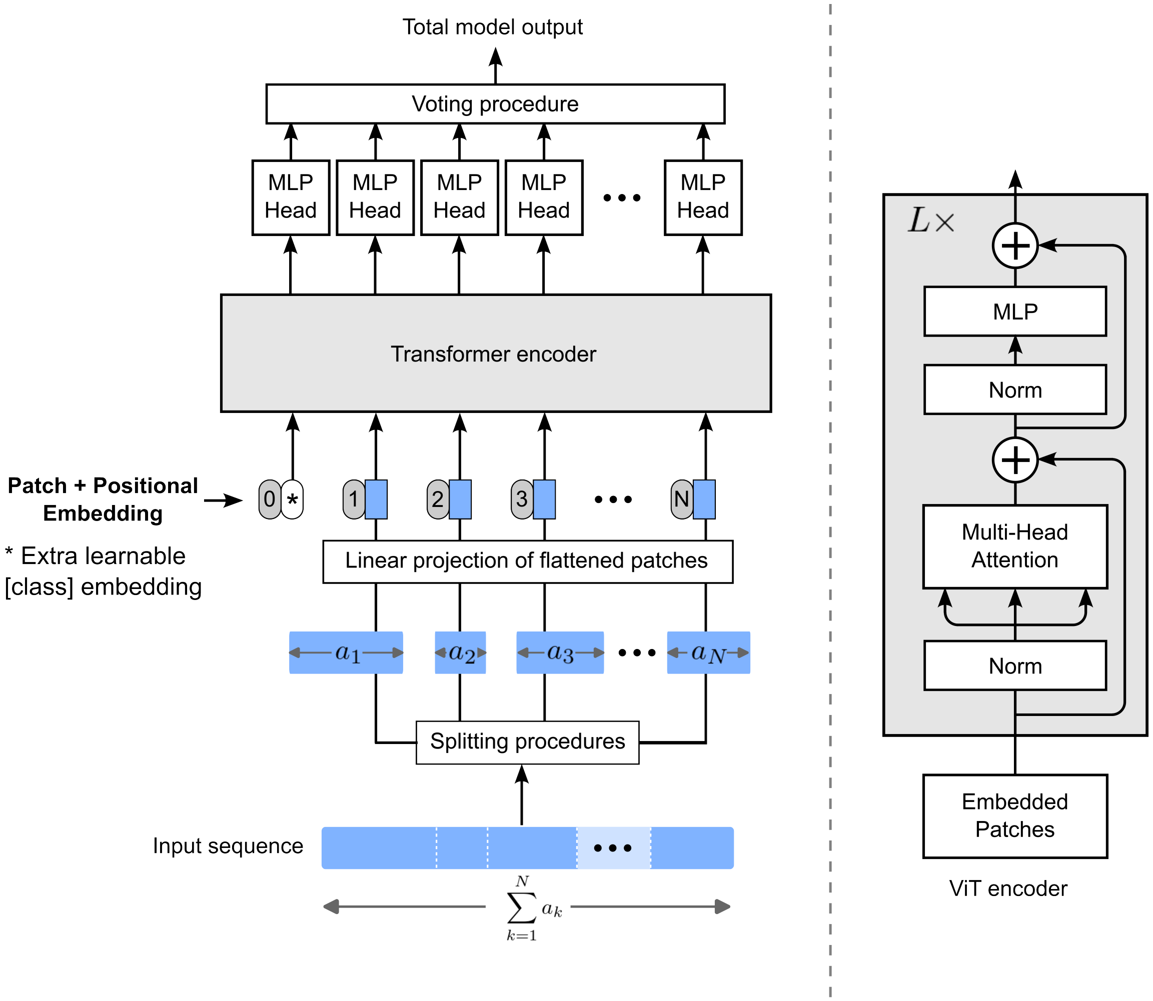

Figure 1: Scheme of the variable vViT. The vViT receives input and split-sequence $\{a_n\}, 1 \leq n \leq N$. The length of the input sequence is $\sum_{i=1}^{N} a_i$. The split sequences are then input to each sector with length of $a_1, a_2, \cdots, a_N$. Each sequence is input into the Transformer encoder after concatenating the positional embedding. Inspired by the original ViT encoder, Transformer is composed of a normlayer (Norm), a multi-head attention layer, and a multilayer perceptron (MLP). Finally, MLP head and voting procedures are performed and the total model output is obtained.
